# Longitudinal associations between problematic TikTok use and academic performance among Chinese college students: mediation by academic burnout and disordered eating

**DOI:** 10.1186/s40337-026-01594-x

**Published:** 2026-05-12

**Authors:** Xiangdong Yan, Shishan Wang

**Affiliations:** 1 College of Electronic and Information Engineering, Shandong University of Science and Technology, Qingdao, China; 2 UCL Institute of Education, University College London, London, UK

**Keywords:** Problematic TikTok use, Disordered eating, Academic performance, GPA, College students

## Abstract

**Purpose:**

Although problematic TikTok use (PTTU) and disordered eating have each been linked to poorer academic outcomes, few studies have examined their interplay—particularly the distinct roles of thinness- versus muscularity-oriented eating—and the contribution of academic burnout. This study tested whether baseline PTTU prospectively predicted subsequent-semester GPA through parallel and sequential pathways involving academic burnout and both eating-pathology orientations among Chinese undergraduates.

**Methods:**

TikTok-using students from three Chinese universities (*N* = 475; M_age_ = 19.34, SD = 1.30) completed the Problematic TikTok Use Scale, Academic Burnout Scale, 12-item EDE-QS, and Muscularity-Oriented Eating Test at semester start (September 2024). At the beginning of the following semester (March 2025), official GPA records were retrieved from university academic systems (with consent) for 431 participants from the baseline cohort. Pearson correlations assessed bivariate associations, and a latent-variable mediation model was estimated in R (v4.1.4) using the lavaan package with robust maximum likelihood (MLR) to derive direct and indirect effects.

**Results:**

Baseline PTTU was inversely associated with later GPA (*r* = − 0.33, *p* < 0.001). Mediation analyses indicated that academic burnout (β = −0.02, 95% CI [− 0.043, − 0.003]), thinness-oriented disordered eating (β = −0.04, 95% CI [− 0.073, − 0.001]), and muscularity-oriented disordered eating (β = −0.03, 95% CI [− 0.061, − 0.002]) each significantly conveyed portions of the PTTU–GPA association, consistent with parallel indirect effects.

**Conclusion:**

PTTU was associated with lower end-of-semester GPA, in part through academic burnout and dual (thinness- and muscularity-oriented) disordered-eating pathways. Integrated campus strategies addressing digital overuse, body-image pressures, and study-related exhaustion may help safeguard academic achievement.

## Abstract (plain language summary)

Many college students use TikTok frequently, and some may find it difficult to control their use. This study examined whether problematic TikTok use was linked to later academic performance among Chinese college students, and whether academic burnout and disordered eating helped explain this link.

We found that students who reported more problematic TikTok use tended to have lower GPA in the following semester. This link was partly explained by greater academic burnout and by disordered eating concerns related to both thinness and muscularity.

These findings suggest that universities should consider problematic short-video use, study-related exhaustion, and body-image or eating-related problems when supporting students’ academic achievement and well-being.

## Introduction

Social media use has become ubiquitous worldwide, reshaping how people communicate, entertain, and learn. Short-video platforms, in particular, have surged in prominence and scholarly attention [35]. TikTok has become one of the most widely used short-video platforms worldwide, attracting substantial engagement across diverse age groups (https://datareportal.com/essential-tiktok-stats). Its endless stream of highly tailored, fast-paced clips appeals to users who favors “bite-sized” entertainment [55]. Yet these same design features have raised concerns about problematic TikTok use (PTTU), characterized by compulsive engagement, distress when restricted, and continued use despite negative consequences, such as heightened loneliness [23, 56]. Such concerns are particularly salient for adolescents and emerging adults, who remain vulnerable to socio-emotional harms linked to excessive short-video consumption [44, 48].

In China’s exam-centered higher-education system, GPA influences scholarships, postgraduate admission, and labor-market prospects [8]. Within this high-stakes context, academic burnout—a syndrome of emotional exhaustion, cynicism toward study, and reduced academic efficacy—has become prevalent [34]. Many students turn to TikTok for momentary “escapes” [65]. While micro-breaks may feel restorative, heavy short-video use risks fragmenting attention, delaying bedtimes, and displacing study time [14, 45]. Early longitudinal evidence in Chinese samples further links higher PTTU with greater academic procrastination [36]. Yet rigorous longitudinal tests of the mechanisms connecting PTTU to academic performance remain limited.

### Problematic TikTok Use (PTTU) and academic performance

Emerging research has begun to examine associations between PTTU and academic-related outcomes. For example, longitudinal research has shown that PTTU predicts increased academic procrastination over time, particularly among male students [36]. However, longitudinal evidence directly linking PTTU to objective academic performance indicators such as GPA remains limited. The Strength Model of Self-Control conceptualizes self-regulation as a finite resource taxed by repeated impulse inhibition [6]. According to this model [6], when individuals exert self-control to resist temptations, their subsequent capacity for effortful regulation may be reduced, increasing susceptibility to distraction and short-term reward seeking. In the context of TikTok use, the platform’s infinite scroll repeatedly confronts students with the decision to continue watching or disengage and return to coursework [14]. Over time, these repeated acts of inhibition may cumulatively tax executive resources required for sustained, goal-directed study [36, 45]. Such resource depletion may manifest in reduced persistence, impaired attentional control, and diminished time-management capacity—behavioral mechanisms directly relevant to academic performance. Building on this framework, we examine whether baseline PTTU is prospectively associated with later academic performance among Chinese undergraduates.

### Mediating role of academic burnout

Academic burnout is the canonical domain-specific manifestation of sustained self-regulatory taxation in learning settings [1]. Drawing on the strength model of self-control, which posits that regulatory resources are limited and susceptible to depletion following repeated exertion [6, 24], continuous inhibition of the urge to keep scrolling, alongside task switching and delay of gratification, is expected to dampen perceived efficacy, heighten exhaustion, and foster cynicism—the core facets of burnout [24, 63]. PTTU characterized by compulsive engagement and difficulty disengaging [56], may intensify these regulatory demands by increasing the frequency of attentional shifts and self-control efforts required during study. Academic burnout has direct behavioral consequences for concentration, persistence, and task engagement, offering a proximal pathway by which PTTU-related depletion translates into reduced quality and quantity of study and, ultimately, lower GPA [24, 63]. Experimental research has demonstrated that repeated self-regulatory exertion impairs subsequent persistence and goal-directed performance [60]. In this study, individuals who had previously exerted self-control performed worse on subsequent persistence and stamina tasks, unless regulatory resources were replenished by positive affect. These findings suggest that repeated acts of self-control may temporarily reduce the capacity for sustained effort. Given that academic burnout is characterized by diminished persistence, emotional exhaustion, and reduced academic engagement, repeated self-regulatory depletion in the context of compulsive TikTok use may gradually contribute to burnout-related processes. In a high-stakes, exam-centered system—where GPA has outsized consequences—burnout is both prevalent and prognostic [34, 36]. Accordingly, we expect academic burnout to partially transmit the prospective association between PTTU and later academic performance.

### Mediating role of disordered eating

Disordered eating encompasses a range of irregular behaviors (e.g., restrictive dieting, binge eating, purging, emotional eating) that do not meet diagnostic thresholds yet carry significant functional costs [57]. Importantly, even disordered eating has been linked to impaired concentration, increased stress reactivity, sleep disturbance, and reduced academic persistence among college students [11, 62, 66], suggesting that its impact extends beyond physical health to core academic functioning. Beyond thinness-oriented disordered eating, contemporary perspectives recognize muscularity-oriented disordered eating as a parallel dimension characterized by distinct behavioral expressions (e.g., compulsive bulking, excessive protein intake, rigid body-enhancement practices) and partially different psychological and sociocultural drivers [27, 28, 29]. Whereas thinness-oriented pathology is typically linked to weight concerns and internalization of slender ideals, consistent with sociocultural and objectification frameworks [5], muscularity-oriented pathology is more closely associated with strength- and performance-based appearance ideals and masculine body norms [39].

PTTU may cultivate conditions conducive to appearance-focused pathology: algorithmic curation disproportionately surfaces body-ideal content (hyper-idealized physiques, “what-I-eat-in-a-day,” rapid transformations), intensifying upward social comparison and ideal internalization [15, 53, 67]. Given TikTok’s highly visual and body-centered content ecology, appearance comparison processes may be particularly salient on this platform, rendering eating-related coping behaviors a theoretically relevant proximal mediator in the pathway from PTTU to academic performance. When self-control resources are already taxed by compulsive scrolling, students may struggle to regulate impulses and adopt short-term mood-repair strategies (e.g., restrictive dieting or compulsive bulking) at the expense of longer-term functioning.

Consistent with this mechanism, engagement with appearance- and eating-related TikTok content has been linked to greater eating disorder symptoms via increased algorithmically recommended appearance-focused content and heightened upward social comparison [15]. Additionally, large-scale evidence suggests that TikTok’s personalization algorithms may disproportionately deliver body-ideal and dieting content to vulnerable users, potentially exacerbating eating disorder symptoms [22]. Research further indicates that physique-focused content is highly prevalent on TikTok, with a substantial proportion of posts centering on diet, exercise, and body ideals [51]. Given TikTok’s visually driven and body-centered content ecology, exposure to strength- and performance-based ideals may reinforce muscularity-oriented disordered eating, supporting its inclusion alongside thinness-oriented pathology.

In China, muscularity concerns have risen alongside thinness pressures among both men and women [27, 28]. In addition, binge eating and purging behaviors have been associated with sleep disturbance, cognitive inflexibility, depressive symptoms, and poorer academic functioning [53]. Distinguishing these two orientations enables a more nuanced test of the mechanisms through which PTTU may differentially affect academic performance. Thus, we posit parallel mediation of the PTTU–GPA association by thinness-oriented and muscularity-oriented disordered eating.

### Chain mediation

The mechanisms may also operate in sequence. PTTU-related resource depletion is likely to present first as academic burnout; ensuing emotional exhaustion and reduced academic efficacy can prompt immediate mood regulation or control-seeking through appearance-focused eating behaviors—strategies offering short-term relief but longer-term academic costs [12]. Empirical work identifies burnout/stress as a prospective predictor of disordered eating among undergraduates [7, 9, 33]. For example, a 6–8-week follow-up study found significant differences in emotional eating across levels of academic burnout, with higher burnout associated with greater thinness-oriented disordered eating [32]. Although direct evidence linking burnout to muscularity-oriented pathology remains limited, recent meta-analytic and empirical research indicates that difficulties in emotion regulation are robustly associated with disordered eating across populations [47, 69]. Accordingly, burnout-related emotional exhaustion and diminished self-regulatory capacity may heighten vulnerability to both thinness- and muscularity-oriented eating pathology. Thus, once entrenched, disordered eating consumes time/energy, disrupts sleep and concentration, and competes with study, further undermining GPA. Accordingly, we test chain-mediated pathways whereby PTTU → burnout → (thinness- or muscularity-oriented) disordered eating → GPA.

### Research gap

Despite expanding interest in short-video platforms, gaps persist: (a) cross-sectional designs dominate and often rely on self-estimated grades; (b) eating pathology is rarely differentiated into thinness- and muscularity-oriented forms, and their parallel vs. sequential roles are under-tested; (c) resource-depletion cascades are more often invoked than empirically examined outside Western contexts; (d) Chinese undergraduate samples remain underrepresented; and (e) cultural specificity in pathway strength and expression is underexplored.

Guided by the resource-depletion and self-regulation framework, we conducted a two-wave longitudinal study of Chinese undergraduates to test whether baseline PTTU is prospectively associated with subsequent semester GPA, and whether academic burnout, thinness-oriented disordered eating, and muscularity-oriented disordered eating mediate this association in parallel and in sequence.

H1 Higher baseline PTTU will be negatively associated with later GPA.

H2a–H2c Academic burnout (H2a), thinness-oriented disordered eating (H2b), and muscularity-oriented disordered eating (H2c) will each partially mediate the PTTU → GPA association.

H3a: PTTU → burnout → thinness-oriented disordered eating → GPA.

H3b: PTTU → burnout → muscularity-oriented disordered eating → GPA.

**Fig. 1:**
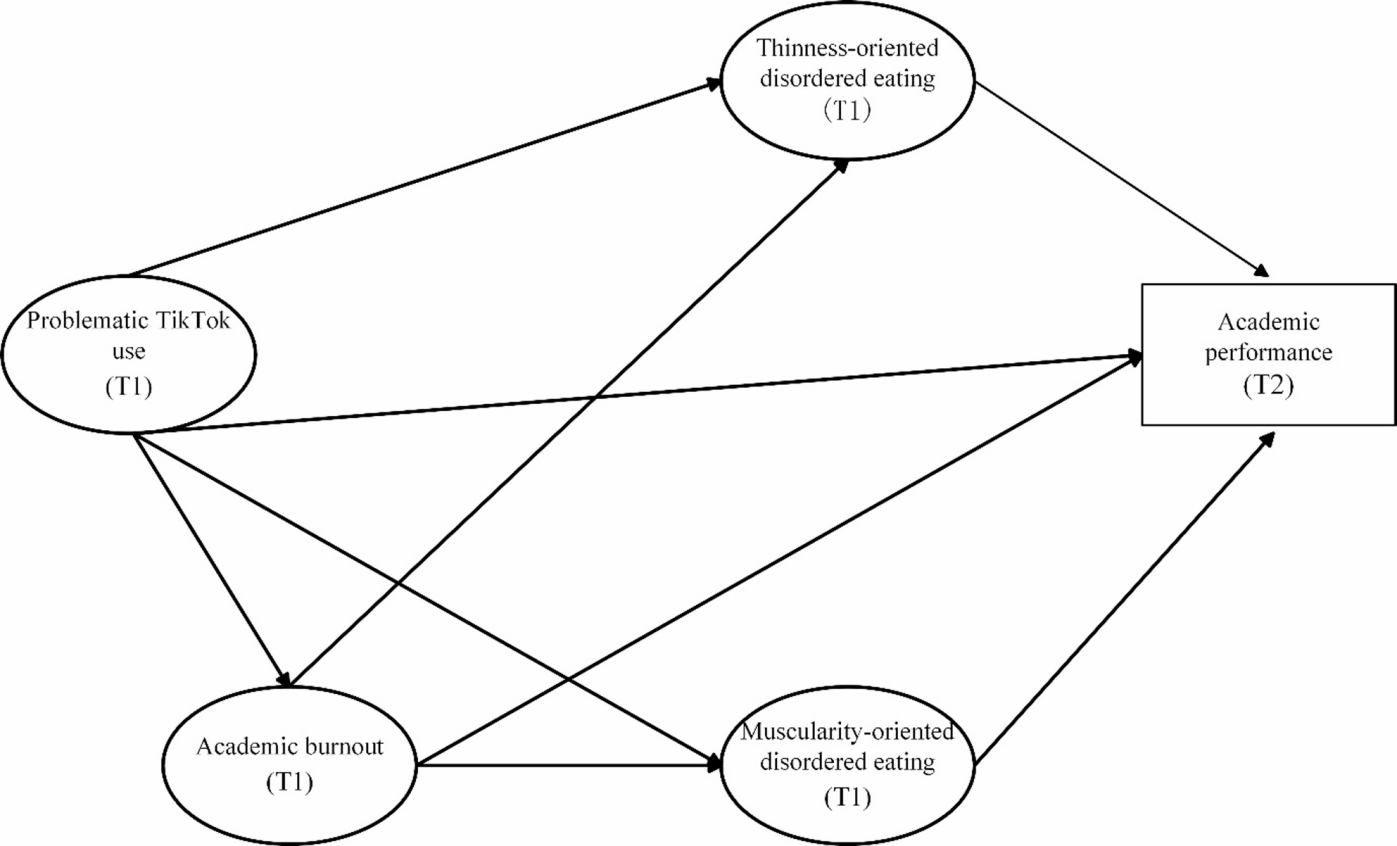
The Conceptual Model Examined in this Study. T1, time 1; T2, time 2

H3c: PTTU → burnout → thinness- and muscularity-oriented disordered eating (in sequence) → GPA. (see Fig. 1).

## Methods

### Sample recruitment and data collection

Baseline data were collected at the start of the semester from Chinese undergraduates at three comprehensive universities located in Shandong (September 2024). Using the Wenjuanxing platform (www.wjx.cn), participants completed measures of PTTU, thinness- and muscularity-oriented disordered eating, and academic burnout. Student affairs counselors at each university distributed the survey link to enrolled undergraduates, and each IP address was restricted to a single submission. The survey opened with an online informed-consent page. To ensure data quality, two attention-check items were embedded; participants who answered both correctly received ¥15 upon completion. Of the 800 students who opened the survey, 475 TikTok users aged 18–24 remained after excluding cases that failed attention checks or reported no TikTok use (154 males, 32.4%; 321 females, 67.6%). Among them, 166 (34.9%) were freshmen, 119 (25.1%) were sophomores, 145 (30.5%) were juniors, and 45 (9.5%) were seniors.

At the beginning of the subsequent semester (March 2025), and with participants’ permission for administrative record linkage, student affairs counselors at each university retrieved official GPA records for the preceding term from the academic information systems and transmitted them to the research team in a secure format. These GPA records reflected students’ final examination results for that semester. This procedure yielded complete GPA data for 431 students from the baseline cohort. GPA was calculated on a 0–4 scale in accordance with the grading system used by these universities, where 4.0 represents the highest academic distinction. The mean GPA was 2.81 (SD = 0.72), corresponding to a mid-range level of academic performance under this 4-point grading system.

### Measurement of problematic TikTok use

We assessed problematic TikTok use (PTTU) with the six-item unidimensional Problematic TikTok Use Scale (PTTUS) [56], adapted from the Bergen Social Media Addiction Scale [4]. In line with prior adaptations, references to “Facebook” were replaced with “TikTok” to capture platform-specific use. A sample item is, “How often during the last year have you felt an urge to use TikTok more and more?” Participants responded to each item on a 5-point Likert scale (0 = “very rarely” to 4 = “very often”), producing a total score from 0 to 24, with higher values indicating greater addictive tendencies. The original validation study of the PTTUS reported a Cronbach’s α of 0.84 [56], and in the present study the scale demonstrated high internal reliability (Cronbach’s α = 0.89). The confirmatory factor analysis indicated good model fit, χ²(9) = 17.40, CFI = 0.994, TLI = 0.990, RMSEA = 0.044, and SRMR = 0.027.

### Measurement of burnout

The Academic Burnout Scale was used to assess academic burnout among university students [34]. This instrument has been shown to be reliable in the Chinese context [64]. It comprises three dimensions: study-related depression (8 items), academic misconduct (6 items), and diminished sense of achievement (6 items), for a total of 20 items. Example statements include “Unit assessment makes me feel disgusted,” “I fall asleep during lectures,” and “I feel the knowledge acquired from courses is useless.” All items were rated on a 5-point Likert scale (1 = completely inconsistent, 5 = fully consistent). In the present study, the Cronbach’s α values were 0.82 for study-related depression, 0.88 for academic misconduct, 0.83 for diminished sense of achievement, and 0.83 for the overall scale.

### Measurement of thinness-oriented disordered eating

Thinness-oriented disordered eating was measured using the 12-item unidimensional Eating Disorder Examination Questionnaire Short (EDE-QS) [20]. This self-report instrument employs a 4-point scale (0–3), with item scores summed to yield a total score ranging from 0 to 36, where higher scores indicate greater symptom severity. A sample item is, “Have you felt fat?”. The Chinese adaptation of the EDE-QS has demonstrated strong psychometric properties among young Chinese populations [29]. Prior validation studies of the EDE-QS have demonstrated good internal consistency in Chinese samples (Cronbach’s α = 0.89) [29]. In the present study, the scale exhibited good internal consistency (Cronbach’s α = 0.88).

### Measurement of muscularity-oriented disordered eating

The Muscularity-Oriented Eating Test (MOET) was developed to assess muscularity-oriented disordered eating behaviors [39]. The 15-item unidimensional scale is rated on a 5-point Likert scale (0 = “never true” to 4 = “always true”), with item scores averaged so that higher mean scores indicate greater symptom severity. An example item is, “I have used meal replacement supplements when I felt full.” The MOET has been validated for use among Chinese young adults [27, 28]. Prior validation studies of the EDE-QS have demonstrated good internal consistency in Chinese samples (Cronbach’s α = 0.84 [28]. In the present study; it demonstrated excellent internal consistency (Cronbach’s α = 0.92).

### Data analysis

All analyses were run in R 4.1.4 using lavaan. Because the survey platform required an answer to every item, the dataset contained no item-level missingness. We first computed Pearson correlations to examine bivariate associations, interpreting effect sizes as small (*r* = .10), medium (*r* = .30), or large (*r* = .50) [10]. Structural equation models were estimated with robust maximum likelihood (MLR), which yields robust standard errors and 95% confidence intervals [40]. Model fit was evaluated with χ²/df, Tucker–Lewis Index (TLI), Comparative Fit Index (CFI), Standardized Root Mean Square Residual (SRMR), and Root Mean Square Error of Approximation (RMSEA). Fit was deemed acceptable when TLI and CFI ≥ 0.90 and SRMR and RMSEA ≤ 0.10 [50]. Based on prior research, BMI, age, and sex may influence both PTTU and academic performance; therefore, these variables were included as controls in the present analyses [2, 17, 18, 46, 61]. Missing data were handled using the full information maximum likelihood (FIML) method to minimize potential bias and retain statistical power. We conducted an a priori Monte Carlo power analysis in R (lavaan) for the proposed chain mediation SEM with covariates. Using the full measurement and structural specification, 1000 datasets were simulated at *n* = 475 and analyzed with MLR. Power, defined as the proportion of significant serial indirect effects (α = 0.05), was 0.80 [0.77, 0.82] and 0.82 [0.79, 0.84], indicating adequate power to detect the hypothesized chain mediation effects.

## Results

The mean age of this sample was 19.34 years [SD = 1.30]. Body mass index (BMI) was determined based on self-reported height and weight values (mean = 20.56, SD = 2.63; range: 14.88–28.41 kg/m^2^). Independent-samples t tests indicated significant sex differences in EDE-QS, *t*(473) = − 2.41, *p* = .016, Cohen’s d = 0.24. Female participants (M = 8.87, SD = 6.42) reported higher levels than male participants (M = 7.40, SD = 5.83). A similar pattern was observed for MOET, *t*(473) = − 2.40, *p* = .017, Cohen’s d = 0.24, with females (M = 1.39, SD = 0.43) scoring higher than males (M = 1.29, SD = 0.39).

Table 1 presents the updated descriptive statistics and bivariate correlations. As shown, PTTU was positively associated with thinness-oriented disordered eating (*r* = .25, *p* < .001; small-to-medium), muscularity-oriented disordered eating (*r* = .23, *p* < .001; small-to-medium), and academic burnout (*r* = .21, *p* < .001; small-to-medium). In turn, GPA was inversely related to PTTU (*r* = –.33, *p* < .001; medium), thinness-oriented disordered eating (*r* = –.28, *p* < .001; small-to-medium), muscularity-oriented disordered eating (*r* = –.27, *p* < .001; small-to-medium), and academic burnout (*r* = –.25, *p* < .001; small-to-medium).

**Fig. 2:**
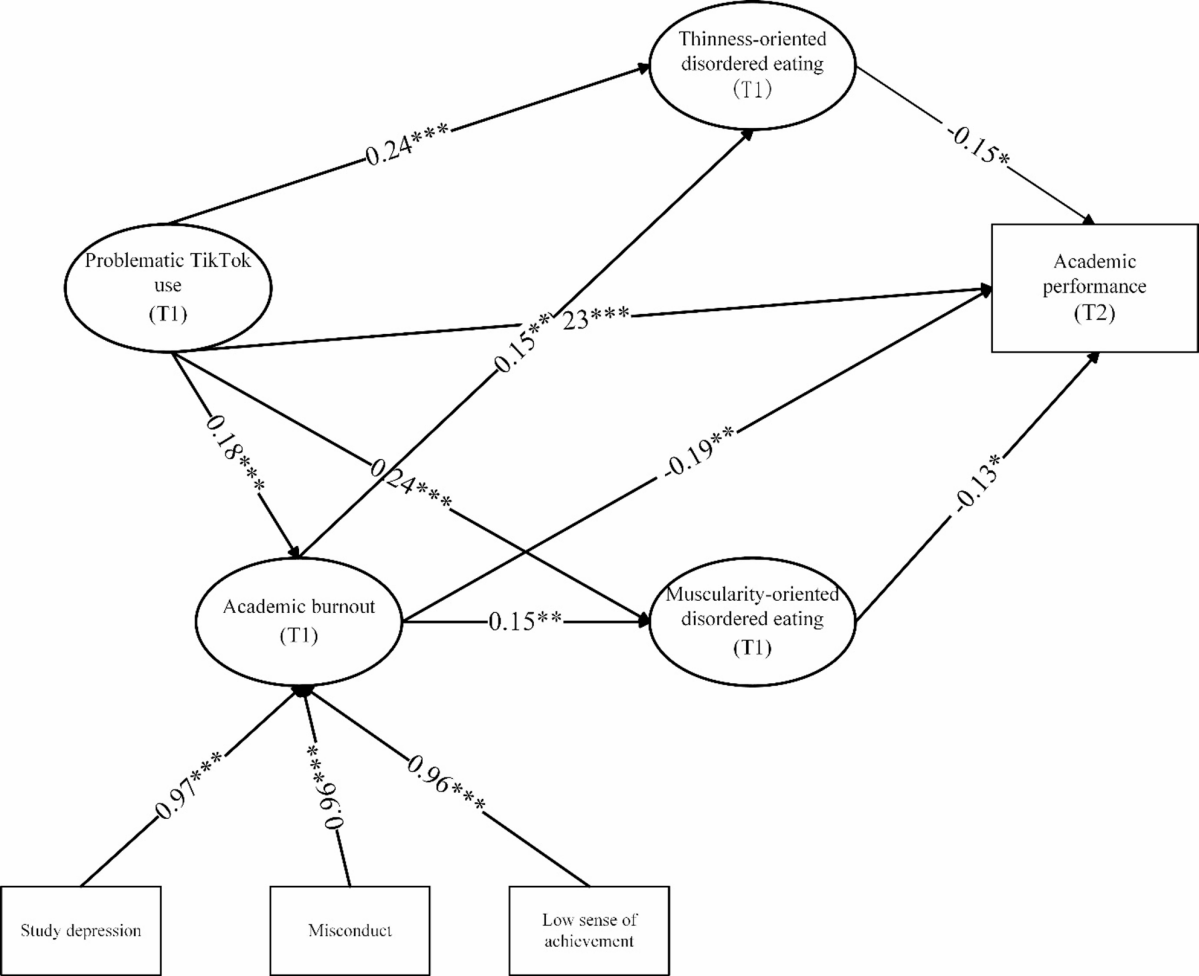
Path Coefficients for the Conceptual Model. The body mass index, age and sex served as covariates in the model. ^*^ *p* < .05, ^**^ *p* < .01, ^***^ *p* < .001. T1, time 1; T2, time 2

As depicted in Table 2; Fig. 2, after controlling for BMI, age, and sex, PTTU was positively associated with academic burnout (*β* = 0.18, *p* < .001), thinness-oriented disordered eating (*β* = 0.24, *p* < .001) and muscularity-oriented disordered eating (*β* = 0.24, *p* < .001), and was directly related to poorer later academic performance (*β* = − 0.14, *p* = .002).

Academic burnout, in turn, predicted higher thinness-oriented disordered eating (*β* = 0.15, *p* = .006), higher muscularity-oriented disordered eating (*β* = 0.15, *p* = .004) and lower academic performance (*β* = − 0.19, *p* = .005). Both forms of disordered eating were themselves linked to lower academic performance (thinness-oriented: *β* = − 0.15, *p* = .020; muscularity-oriented: *β* = − 0.13, *p* = .012).

Mediation testing revealed a significant overall transmission of the impact of PTTU on subsequent academic performance through maladaptive eating and burnout processes (*β* = − 0.08, 95% CI [–0.144, − 0.054]). The largest portions of this total effect travelled through the single-step routes involving thinness-oriented disordered eating (*β* = − 0.04, 95% CI [–0.073, − 0.001]) and muscularity-oriented disordered eating (*β* = − 0.03, 95% CI [–0.061, − 0.002]). A further, but smaller, contribution arose from the direct pathway via academic burnout (*β* = − 0.02, 95% CI [–0.043, − 0.003]). The two three-step chains—PTTU → academic burnout → thinness-oriented disordered eating → academic performance (*β* = − 0.004, 95% CI [–0.009, 0.001]) and PTTU → academic burnout → muscularity-oriented disordered eating → academic performance (*β* = − 0.003, 95% CI [–0.009, 0.001])—did not reach statistical significance, indicating that the incremental “burnout → disordered eating” step adds little beyond the simpler one-step mediation paths. Taken together, the results show that PTTU lowers academic performance both directly and indirectly via higher academic burnout, greater thinness-oriented disordered eating, and greater muscularity-oriented disordered eating. But the chain mediations are not significant (see Table 3 for full details).

## Discussion

This study extends the literature on problematic short-video use by demonstrating that PTTU was associated with lower end-of-semester academic performance among Chinese undergraduates, primarily through three parallel pathways—academic burnout, thinness-oriented disordered eating, and muscularity-oriented disordered eating. These findings support our integrated model and confirm the proposed hypotheses. Importantly, they underscore the need for targeted interventions that address both excessive social media use and body-image-related concerns to enhance students’ academic outcomes and overall well-being.

The medium negative correlation between PTTU and GPA (*r* = –.33) aligns with effect sizes previously reported for excessive gaming and general social media addiction, underscoring the academic costs of persistent short-video engagement. Two complementary mechanisms may account for this association. Cognitive-load theory [59], predicts that an endless stream of dynamic, algorithm-curated clips taxes working memory [59], interrupts deep processing, and encourages multitasking—processes known to erode learning efficiency [21, 70]. Self-regulation depletion theory, adds that the repeated need to suppress the urge to continue scrolling drains executive resources, leaving fewer reserves for sustained study or disciplined time management [6]. Together, these frameworks elucidate why academic burnout emerges as a key mediator in the PTTU–GPA relationship.

In addition, both thinness-oriented disordered eating (*r* = –0.28, *p* < 0.001) and muscularity-oriented disordered eating (*r* = –0.27, *p* < 0.001) were moderately negatively correlated with GPA. From a self-regulatory perspective, disordered eating reflects sustained goal pursuit centered on body modification, requiring continuous monitoring of diet, appearance, and behavioral routines [6]. Such persistent body-focused striving competes with academic goals for limited cognitive and behavioral resources. Importantly, muscularity-oriented disordered eating represents a structured, performance-driven investment in physique enhancement, often involving rigid dietary control, supplementation, and intensive training schedules [38, 39]. These highly regulated routines demand substantial planning, time allocation, and psychological commitment, thereby directly conflicting with academic responsibilities. Accordingly, both thinness- and muscularity-oriented patterns can be understood as competing self-regulatory systems that impose comparable cognitive and behavioral costs on academic functioning.

Our findings indicate that thinness-oriented disordered eating significantly mediates the association between PTTU and GPA. Previous studies have reported bivariate links among problematic TikTok use, thin-ideal internalization, and poorer academic outcomes [16, 30, 68], the present results extend this evidence by elucidating the underlying mechanism. Repeated exposure to appearance-focused content likely amplifies body-shape concerns and restrictive eating behaviors, which—through heightened stress, disrupted time management, and reduced concentration—ultimately impair academic performance. This sequence aligns with self-regulation depletion theory, suggesting that the cognitive effort required to sustain dietary restraint draws upon the same executive resources essential for complex learning and academic persistence.

A similar pattern emerged on the muscularity side. Consistent with research on TikTok’s “fitspiration” culture [25, 54], muscularity-oriented disordered eating also transmitted PTTU’s academic costs. Behaviors such as high-protein over-supplementation, rigid meal plans, and time-intensive training regimens can crowd out study hours, disrupt nutritional balance, and heighten anxiety or depressive symptoms [13, 39]. These psychological and physiological burdens likely manifest as attentional lapses and motivational deficits that undermine coursework [3, 19, 38]. These adverse effects likely stem from the distractions and motivational deficits associated with eating disorders among college students [31, 42]. Importantly, the indirect effect sizes for thinness- and muscularity-oriented eating were statistically equivalent in our model, suggesting that effective interventions must address the full spectrum of appearance-based pressures rather than focusing solely on the thin ideal. Evaluations of PTTU’s impact on student achievement should therefore incorporate multiple disordered-eating phenotypes to capture the breadth of these risk pathways.

A more plausible interpretation of the null sequential effect involves variance overlap and competing pathways rather than the six-month lag itself. Such discrepancies between significant component paths and nonsignificant overall indirect effects are not uncommon in mediation models, particularly in latent-variable or longitudinal designs, where small effect sizes, shared variance, or limited statistical power may attenuate the total indirect estimate [26, 37].

Problematic TikTok use likely already captures much of the psychosocial strain that fuels maladaptive coping; once the direct PTTU → disordered-eating pathway is accounted for, academic burnout contributes little unique variance. This pattern fits the dual-pathway framework of body-image disturbance [58]: an appearance-comparison pathway operates alongside a negative-affect (stress) pathway. In a feed saturated with idealized bodies, the comparison pathway is likely to dominate, rendering the slower negative-affect route (here indexed by burnout) statistically redundant when both are entered simultaneously. Meta-analytic evidence on social-media exposure supports this hierarchy: appearance-comparison cues elicit near-immediate disturbances in body image and eating behavior, whereas mood-driven effects are typically smaller and emerge more gradually [49].

The findings point to the need for multi-level prevention strategies. At the app-design level, platforms could implement optional usage dashboards or timed reminders to interrupt the infinite-scroll loop and encourage mindful disengagement. At the university level, digital-literacy programs could help students identify algorithm-driven comparison traps and establish intentional viewing boundaries. Counseling centers may expand body-image interventions to address both thinness and muscularity ideals, while academic advisors could collaborate with campus fitness clubs to promote balanced exercise and nutrition practices that complement—rather than compete with—academic commitments.

Several limitations warrant mention. First, although GPA was measured longitudinally using official university academic records, it represents only one indicator of academic performance and may not fully capture broader dimensions such as learning engagement, skill acquisition, or qualitative academic achievement. In addition, prior GPA was not controlled in the present study, which may limit causal interpretation. Future research could incorporate more comprehensive and multidimensional indicators of academic functioning, as well as account for prior academic performance to strengthen causal inference. Second, BMI and baseline eating patterns were also self-reported, which may be subject to reporting bias and potential inaccuracies. Third, experimental or ecological momentary assessment designs could strengthen causal inference and capture within-day fluctuations in self-control, mood, and TikTok exposure. In addition, although the present study focused on disordered eating as a theoretically grounded mediator, other potential mechanisms, such as sleep disturbance, attentional fragmentation, or cognitive overload, were not examined and may also contribute to the association between PTTU and academic performance. Future research should incorporate multiple mediating pathways to develop a more comprehensive explanatory model. Fourth, the present study focused exclusively on undergraduate students. Given developmental differences in academic performance and social media engagement [41, 43, 52], future research should examine whether these pathways replicate in adolescent samples. Finally, the present study did not assess objective social media use duration or screen time, which may provide additional contextual information regarding exposure intensity. Future research could incorporate usage frequency or duration metrics to further refine the understanding of how PTTU relates to academic performance.

In sum, this two-wave study study elucidates how PTTU contributes to lower academic performance among Chinese undergraduates. Leveraging temporal ordering—assessing PTTU, academic burnout, and thinness- and muscularity-oriented disordered eating at baseline, and GPA in the following term—we found that higher PTTU was associated with lower end-of-semester GPA. Mediation analyses supported complementary mechanisms consistent with resource-depletion, cognitive-load, and social-comparison processes: academic burnout, thinness-oriented disordered eating, and muscularity-oriented disordered eating each accounted for unique portions of the PTTU–GPA association. Collectively, these findings advance a multi-level framework—from platform design and algorithmic curation to individual self-regulation—on which educators, clinicians, and policymakers can base evidence-informed strategies (e.g., digital-hygiene training, burnout mitigation, and body-image literacy initiatives) to foster healthier media use and protect students’ academic trajectories.

**Table 1:** Means, standard deviations, and Pearson correlation for the study variables (*n* = 431)

Variable	1	2	3	4	5	6	7
1 PTTUS (T1)	–						
2 MOET (T1)	0.23***	–					
3 EDE-QS (T1)	0.25***	0.39***	–				
4 Academic Burnout (T1)	0.21***	0.18***	0.19***	–			
5 GPA (T2)	–0.33***	–0.27***	–0.28***	–0.25***	–		
6 Age (T1)	0.20***	0.01	0.01	0.11*	–0.26***	–	
7 BMI (T1)	–0.05	0.12**	0.12**	0.06	–0.07	–0.04	–
M	9.6	1.36	8.39	59	2.81	19.34	20.56
SD	5.86	1.27	6.27	24.18	0.72	1.30	2.63

PTTUS: Problematic TikTok Use Scale; EDE-QS: 12-item Eating Disorder Examination Questionnaire; MOET: Muscularity-Oriented Eating Test; BMI: body mass index; M: mean; SD: standard deviation; T1, time 1; T2, time 2

^*^p < .05; ^**^p < .01; ^***^ *p <* .001

**Table 2:** Regression coefficients and standard errors from the mediation model

Antecedent variable	Academic burnout (T1)			Thinness-oriented disordered eating (T1)			Muscularity-oriented disordered eating (T1)			Academic performance (T2)		
	β	95% CI	SE	β	95% CI	SE	β	95% CI	SE	β	95% CI	SE
Sex	0.06	−0.04–0.15	0.05	0.13 *	0.05–0.23	0.04	−0.11*	−0.21 – −0.01	0.05	−0.03	−0.17–0.11	0.07
Age	0.08	−0.01–0.17	0.04	−0.05	−0.15–0.05	0.05	0.04	−0.06–0.13	0.06	−0.04	−0.21–0.13	0.07
BMI	0.09	−0.001–0.18	0.05	0.15**	0.06–0.25	0.05	0.07	−0.02–0.16	0.05	−0.01	−0.11–0.09	0.05
Problematic TikTok Use (T1)	0.18***	0.09–0.27	0.05	0.24***	0.14–0.35	0.05	0.24***	0.14–0.34	0.05	−0.14**	−0.24 – −0.04	0.05
Academic burnout (T1)				0.15**	0.05–0.25	0.05	0.15**	0.05–0.24	0.05	−0.19**	−0.30 – −0.09	0.05
Thinness-oriented disordered eating (T1)										−0.15*	−0.27 – −0.03	0.06
Muscularity-oriented disordered eating (T1)										−0.13*	−0.24 – −0.02	0.06
R²	0.12			0.12			0.11			0.2		

CI: confidence interval; SE: standard error; BMI: body mass index. ^*^ *p* < .05, ^**^ *p* < .01, ^***^ *p* < .001. T1, time 1; T2, time 2

**Table 3:** Two pathways of indirect effects

Indirect effect	Point estimate	95% CI	SE
Total	−0.08	−0.144 – −0.054	0.02
Path 1: PTTUS → TE → AP	**−0.04**	**−0.073 – −0.001**	**0.02**
Path 2: PTTUS → ME → AP	**−0.03**	**−0.061 – −0.002**	**0.02**
Path 3: PTTUS → AB → AP	**−0.02**	**−0.043 – −0.003**	**0.01**
Path 4: PTTUS → AB → TE → AP	−0.004	−0.008–0.001	0.004
Path 5: PTTUS → AB → ME → AP	−0.003	−0.008–0.001	0.003

CI: confidence interval; SE: standard error; PTTUS: Problematic TikTok Use Scale; AB: Academic burnout; TE: thinness-oriented disordered eating; ME: muscularity-oriented disordered eating

## Data Availability

The data will be available from the corresponding author upon request.
